# Impact of HPV vaccination on HPV infection and cervical related disease burden in real-world settings (HPV-RWS): protocol of a prospective cohort

**DOI:** 10.1186/s12889-022-14474-1

**Published:** 2022-11-18

**Authors:** Zhike Liu, Pei Li, Xueyang Zeng, Xiaoying Yao, Yexiang Sun, Hongbo Lin, Peng Shen, Feng Sun, Siyan Zhan

**Affiliations:** 1 Department of Epidemiology and Biostatistics, Peking University School of Public Health, Xueyuan Road No.38, Beijing, 100191 China; 2 Shanghai Municipal Health Commission, Shibocun Road No.300, Shanghai, 200125 China; 3 Yinzhou District Center for Disease Control and Prevention, Xueshi Road No.1221, Ningbo, 315100 China; 4 Institute for Artificial Intelligence, Peking University, Beijing, 100191 China; 5 Research Center of Clinical Epidemiology, Peking University Third Hospital, Haidian District, Beijing, 100191 China

**Keywords:** HPV vaccine, Cervical cancer, Cervical cancer screening, Prospective cohort

## Abstract

**Background:**

Cervical cancer is one of the most common cancers in women and could be prevented by human papilloma virus (HPV) vaccination. *Cervarix*, the first available HPV vaccine, has been widely administrated to Chinese women, while little was known about its effect on the prevention and control for HPV related diseases in China. The study aims to assess the impact of *Cervarix* on HPV infection and cervical related diseases in real world.

**Methods:**

This is a prospective, multi-age birth cohort study to investigate the incidence and continuous status of HPV infection, and relevant cervical diseases by exposure status (with *Cervarix* vaccination history or without any HPV vaccination history). It is planned to recruit 12,118 eligible women at age of 9 to 45 years from vaccination clinics or hospital outpatient clinics, and then follow up them for three years. The standard questionnaire will be used to collect information such as demographic characteristics, menstruation and obstetrical histories, history of sexual behavior, personal behavior history, history of disease and pathogen infection, medication history, and family history at baseline. After three years, the changes of these behaviors will be investigated again, and other related health status information will be retrieved from the electronic health records during the follow-up period. If available physically and legally, the cervical cancer screening will be performed, including type-specific HPV deoxyribonucleic acid (DNA) polymerase chain reaction (PCR) testing and contingent thinprep cytologic test (TCT) and colposcopy. The free cervical cancer screening will be captured and uploaded timely to the Yinzhou Regional Health Information Platform (YRHIP); therefore, the long-term outcomes of participants will be monitored.

**Discussion:**

This prospective cohort study will assess the impact of HPV vaccine on HPV infection and related cervical diseases in women aged 9–45 years, which makes up for the lack of evidence in Chinese women. The results of this study will provide support for understanding the impact of HPV vaccination in China, and make a contribution to increasing HPV vaccination and cervical cancer screening coverage in China.

**Trial registration:**

This study has been retrospectively registered on *clinicaltrials.gov* (NCT05341284) on April 22, 2022.

## Background

Cervical cancer was the fourth most common cancer in women, with approximately 0.60 million cases and over 0.34 million deaths from cervical cancer worldwide in 2020 [1]. China is one of the countries with the most cases contributed with approximately 0.11 million cases and 0.05 million deaths [2]. Further, both the incidence of cervical cancer and disease burden still increased in the last decades, and its onset age becomes much younger in Chinese women [3, 4]. Human Papillomavirus (HPV) infection is the primary cause of cervical cancer [5], specifically, HPV16 and 18 infections accounted for 84.5% of invasive cervical cancer in China [6].

**Fig. 1:**
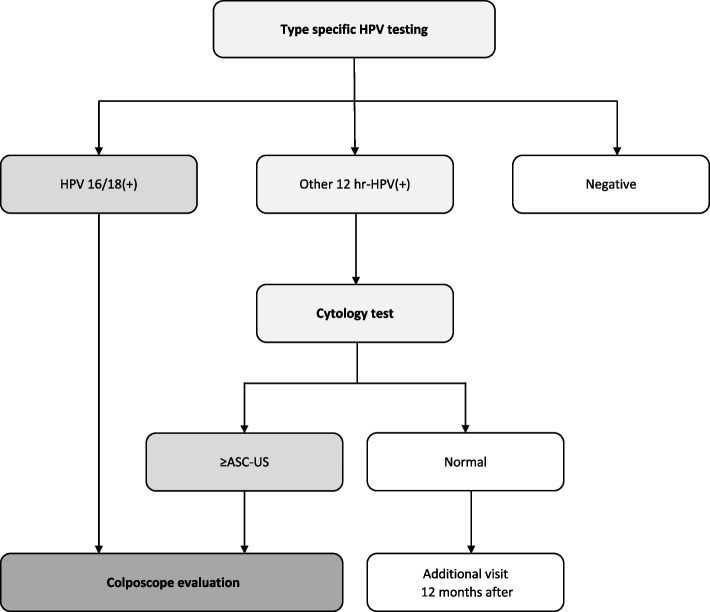
Cervical cancer screening procedure

Expanding the coverage of HPV vaccines is an essential part of the 90–70-90 target proposed by the World Health Organization (WHO) for eliminating cervical cancer [7]. In a modeling study, with 70% coverage of bivalent vaccine, cancer cases can decrease by 58% to 33% in rural areas and 69% to 32% in urban areas at ages of 12 to 55, respectively [8]. China has also been making effort to increase vaccination coverage. Since 2016, HPV vaccines have been introduced for females aged 9–45 in mainland China, such as *Cervarix* (9–45 years old) in 2016, *Gardasil 4* (20–45 years old) in 2017, *Gardasil 9* (16–26 years old) in 2018 and the domestic bivalent HPV vaccine *Cecolin* (9–45 years old) in 2019 [9–12]. These vaccines were not included in the programmed immunization and were paid for by the receivers. Since 2020, some of cities in China have launched free HPV vaccination programs for girls under 14 years, which only included the domestic bivalent HPV vaccine *Cecolin* for now [13, 14]. In addition, China is also popularizing cervical cancer screening to accelerate the elimination of cervical cancer. China has initiated *the Two Cancers (the cervical cancer and breast cancer)* Screening Program in 2009, which provided females aged 35 years and over with cervical cancer and breast cancer screening for free [15]. Screening strategies varied across regions, including HPV-initiated and TCT-initiated tests. As for Yinzhou, Ningbo, where our study will be conducted, the standard cervical cancer screening process initiate with a type-specific HPV DNA PCR testing, and for the ones with HPV infection will be referred to colposcopy or cytology test, while those who are tested negative will be invited for another screening after 3 years (Fig. 1).

Several prospective cohort studies assessed the impact of HPV vaccination, such as a Dutch prospective cohort initiated in 2009 [16], a 10-year prospective cohort study in Greece between 2009–2019 [17], a population-based birth cohort study in Sweden [18], and a register-based cohort study in Denmark [19]. These cohorts consistently provided supportive evidence for the effectiveness of HPV vaccination and its policies. However, there are no such studies conducted in China, where the promotion of HPV vaccines seems much more pressing. *Cervarix,* the first available HPV vaccine for Chinese women, have great acceptance in China [20]. Here, as an exemplar, the prospective cohort study with *Cervarix* assesses the impact of HPV vaccination on HPV infection and cervical related diseases in Chinese women. The indicated population of Cervarix are females aged 9–45, thus, the whole indicated population should be included into the real-world study. In addition, the age distribution of HPV-infected population in China is bimodal (< 25 years and 41–45 years) [21], therefore, it is also important to evaluate the role of HPV vaccination in women aged 41–45 years in China.

Yinzhou, in which this study will be conducted, is a district of Ningbo, Zhejiang province, which is one of the most developed provinces of China. The Center for Disease Control and Prevention (CDC) of Yinzhou set up an integrated electronic health information system for local residents in 2005 [22]. By the year of 2009, this regional system has covered nearly all health-related activities of residents from birth to death. For its high population coverage and superior data quality, this platform is identified as one of the best sources of post-marketing drug surveillance in China. Hence, we choose to conduct our study in this area and use this platform to manage our data, which will allow us to expand the follow-up period in a real-world setting.

### Aims

The study aims to evaluate the impact of the AS04 adjuvanted HPV 16/18 vaccine (Brand name: *Cervarix),* for HPV infection and related cervical diseases in real world, with the following objectives:

1. To describe the incidence of HPV 16/18 infection by exposure status, and to evaluate the effectiveness of *Cervarix* on HPV 16/18 infection in birth cohorts
2. To describe the incidence of cervical lesions or diseases, and other 12 high-risk HPV infection by exposure status in birth cohorts

## Methods

### Study design and setting

Prospective, multi-age birth cohort study to investigate the incidence and its change of HPV infection and relevant cervical disease by exposure status (with *Cervarix* vaccination history or without any HPV vaccination history) in recruited subjects between 9 to 45 years old in Yinzhou District. Participants of this study will be recruited from local vaccination sites and the hospital outpatient clinics, and they will be divided into three age groups (9–20 years, 21–30 years,31–45 years) at baseline. Participants who get vaccination of the first or second dose of the AS04 adjuvanted HPV 16/18 vaccine during the recruitment period will be included in the exposed group, and those who are not inoculated with any HPV vaccines before the recruitment will be included in the non-exposed group. All the participants will be visited twice during the study period, one at baseline and the other after a three-year follow-up. A standard questionnaire and cervical cancer screening will be given at each of the two visits to collect information. This whole study process was designed and will be conducted by School of Public Health, Peking University and the CDC of Yinzhou District.

### Study sample and enrollment

This study will enroll the permanent female residents between 9 to 45 years old in the Yinzhou District, with *Cervarix* vaccination history or without any HPV vaccination history.

Subjects who meet the enrollment criteria will be recruited by site investigators at eligible vaccination sites and hospital outpatient clinics for the study. Based on feasibility assessments and historical vaccination data on the YRHIP, baseline recruitment of subjects is expected to continue for 24 months.

### Inclusion criteria

All subjects must satisfy the following criteria at study entry:

- Permanent female resident in the Yinzhou District (i.e., at least residing in the Yinzhou District for a 3-year period and at least 6 months in every 12-month period).
- Between 9 and 45 years old at time of the enrollment.
- Receiving 1 or 2 doses of *Cervarix* (exposed group); or without any HPV vaccination history (non-exposed group).
- Subjects for whom the investigator believes that they or their parent(s)/ Legally Acceptable Representative(s) (LAR(s)) can and will comply with the requirements mentioned in the protocol (e.g., physically and mentally healthy, be able to complete the baseline and follow-up survey and would comply with the visits, etc.).
- Written informed consent will be obtained from the subject. For subjects who are below the legal age of consent, written informed consent must be obtained from the parent(s)/LAR(s) of the subject and informed assent must be obtained from the subject according to EC requirement as well as local law. Subjects must understand the protocol and be voluntarily willing to join this study with written informed consent form.

### Exclusion criteria

The following criteria should be checked at the time of study entry. If any apply, the subjects must not be included in the study:

- Pregnancy at the enrollment.
- Females with historical cervical diseases (i.e., cervical intraepithelial neoplasia (CIN)1, CIN2, CIN3, and cervical cancer) before the recruitment.
- After hysterectomy.
- Females with malignant tumor history or other severe diseases (e.g., liver failure, heart failure, etc.), whose life expectancy is less than 12 months.
- Females who (1) have historical HPV vaccination, or (2) are in non-exposed groups but have clear intention for HPV vaccination in next 3 years (confirmed by field investigators before enrollment).
- Any high-risk HPV (hr-HPV) DNA (i.e., HPV 16/18/*31/33/35/39/45/51/52/56/58/59/66/68*) positive result at baseline.
- Having abnormal colposcope diagnosis or cervical diseases at the baseline screening (women tested HPV positive or having abnormal colposcope diagnosis or cervical disease will be followed up and managed through the local referral system).

### Drop-out criteria

The following criteria should be checked at or right before the follow-up visit. If any become applicable during the study, the subject will withdraw from the study.

- Pregnancy within the pre-scheduled visiting phase and not eligible for cervical cancer screening.
- Subjects in the non-exposure group who received any HPV vaccine during the study period.
- Death due to causes other than cervical cancer.
- Lost to follow-up (i.e., migrated out the Ningbo city, refusing to respond).

### Sample size

Per the previous research, the effectiveness assumptions for *Cervarix* will be 83%, 66%, and 50% for the cohorts of 9–20, 21–30 and 31–45 years of age respectively [23, 24]. The frequency of exposed group vs. non-exposed group will equal to 1: 1 with the level of significance α = 0.05 and statistic power 1-β = 0.8. The statistical analysis was performed on PASS15.0 (Tests for Two Proportions). Considering the loss to follow-up (5% estimated) and subtraction due to the baseline HPV infection and the contamination during follow-up (25% estimated), there will be 30% loss at the end of study. To mitigate this, the targeting recruitment number will be 1/(1–0.3)*100% of the sample size of effectiveness calculation. The detailed variables and calculation of the sample size is demonstrated in the table below (Table 1).

**Table 1:** Sample size calculation

Age group(y/o)	Incidence of HPV 16/18 infection	Vaccine effectiveness	Frequency ratio	Exposure group	Non-exposure group	Sample size for effectiveness calculation	Targeting recruitment number
9–20	0.02	0.83	1:1	661	661	1322	1890
21–30	0.02	0.66	1:1	1208	1208	2416	3452
31–45	0.02	0.50	1:1	2371	2371	4742	6776
Total	-	-	-	4240	4240	8480	12,118

### Endpoints

#### Primary endpoint

Cervical infection with HPV-16 and/or HPV-18 (by PCR), *defined as at least one positive HPV-16 or HPV-18 DNA PCR assay at enrollment (Month 0) or follow-up (Month 36) or any additional screenings during the study period.*

#### Secondary endpoints

Cervical infection with HPV-16 and/or HPV-18 over 12 months (measured by two tests with an interval of 12 months), *defined as at least one positive HPV-16 or HPV-18 DNA PCR assay at the additional visit in 12 months after getting the positive HPV-16 or HPV-18 test result at enrollment (Month 0) or follow-up (Month 36) or any additional screenings during the study period.*

Cervical infection with 12 other high-risk HPV besides of type 16/18 (by PCR), *defined as at least one positive for HPV 31/33/35/39/45/51/52/56/58/59/66/68 or multiple types DNA PCR assay at enrollment (Month 0) or follow-up (Month 36) or any additional screenings during the study period.*

Any cervical lesions or diseases in birth cohorts, *defined as atypical squamous cells of undefined significance (ASC-US), atypical glandular cells (AGC), atypical squamous cells, cannot exclude for highly squamous intraepithelial lesions (ASC-H), low-grade squamous intraepithelial lesion (LSIL), high-grade squamous intraepithelial lesion (HSIL), Cervical Intraepithelial Neoplasia (CIN) 1, CIN2, CIN3, squamous cell carcinoma (SCC), adenocarcinoma *in situ* (AIS) and adenocarcinoma (ADC). Any hr-HPV type infection is defined as at least one positive for HPV 16/18/31/33/35/39/45/51/52/56/58/59/66/68 or multiple types DNA PCR assay in the aforesaid cervical sample.*

### Study procedure

**Fig. 2:**
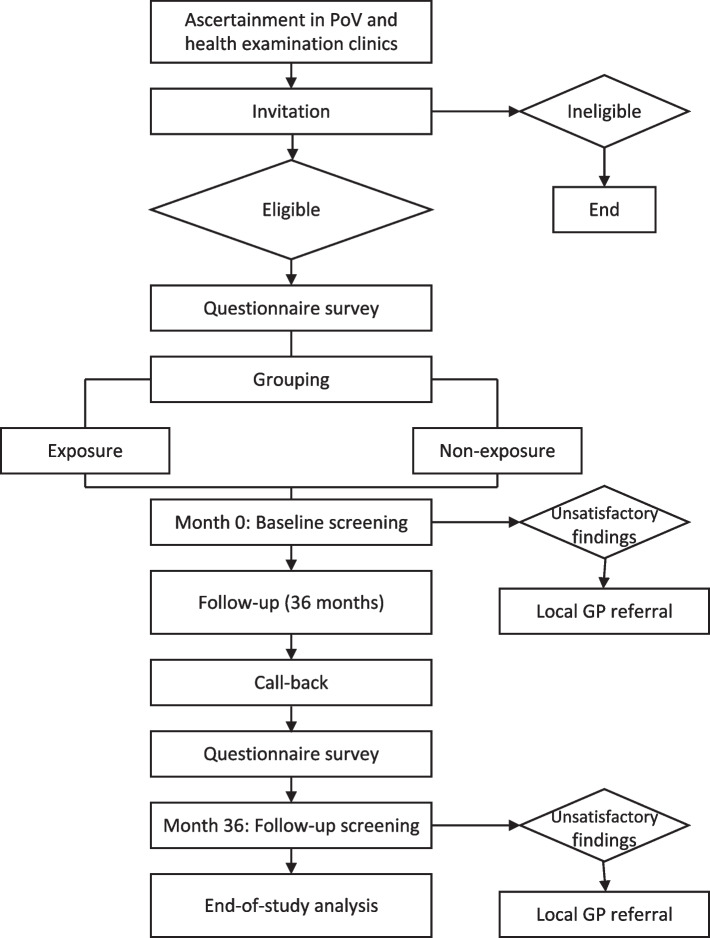
Study procedure

Figure 2 shows the details of the study’s procedure. This study will be conducted in the local vaccination clinic/point of vaccination where *Cervarix* and its administration service are available and health examination clinics of hospitals where the cervical cancer screening is available. The study subjects will be invited and the ones who have satisfied the inclusion criteria will be enrolled by the field investigators including gynecologists and general practitioners in the aforesaid study sites.

Subjects will be stratified into three age groups (9–20 y/o, 21–30 y/o and 31–45 y/o) and divided into exposed and non-exposed groups by vaccination status. The exposed group will include subjects who received 1 or 2 doses of *Cervarix* and the non-exposed group will include subjects who did not receive any doses of HPV vaccine.

During the study period, two visits will be scheduled for all subjects at Month 0 and Month 36, which means the follow-up period will be 3 years. In the first visit, the questionnaire survey will be administrated for all the subjects to obtain the demographic information, sexual history, menstrual and obstetrical histories, etc. For the ones who are eligible for the cervical cancer screening (i, Have sexual debut at the enrollment; ii, no contradiction of screening and iii, are willing to undergo the screening), the type-specific HPV DNA PCR testing will be given within 2 weeks after the enrollment. After 3-years follow-up, the subjects will be called back for the second visit. A questionnaire which measures any changes of behavior during the follow-up period will be performed. Then the second screening with type-specific HPV DNA PCR testing will be given within 2 weeks after the call-back.

At each visit, type-specific HPV DNA PCR testing will be the primary screening approach, and for the ones with unsatisfactory findings (i.e., hr-HPV infection) will be referred to the local abnormal cervical cancer screening results management system for further medical activities (Fig. 1)

By the end of the follow-up period, the prevalence of the HPV infection, cervical disease (e.g., CIN1, CIN2, CIN3, SCC, AIS, etc.) will be described in each birth cohort by their exposure status. If the sample size is sufficient, the effectiveness of *Cervarix* will be assessed as the exploratory objective.

### Loss of follow-up

For those who were lost, retrieve will be conducted by making phone calls as far as possible. For those who failed to be tracked, the reasons for loss to follow-up will be investigated as far as possible, and the reasons for loss to follow-up will be analyzed. At the same time, the baseline data of lost and non-lost patients, and the ratio of loss to follow-up between exposed and non-exposed groups will be compared to estimate the possible bias.

### Data collection

All the data collected from the surveys and cervical cancer screenings will be documented in the YRHIP, (approval No.: GKYBSZ [2020] No. bc0015; Applicant: Ningbo Yinzhou Center for Disease Control and prevention; Approval time: October 2020).

Once the subject is recruited into the study with signed informed consent form, she will be identified in the YRHIP via her resident identification number and marked as the study subject, and new subpage which contains the study data will be created on her personal file in the YRHIP. The data of the paper-based questionnaire survey will be transcribed into corresponding fields and the cervical cancer screening data will be synchronized with existing healthcare activity page on real-time. Besides the survey and screening data, we’ll also obtain the data of general information, enrollment date, scheduled time of second visit, call-back status, HPV vaccination history, self-initiated cervical cancer screening, cervical related treatment or medication from the YRHIP.

### Statistical analysis

We will describe the distribution and group differences of the subjects’ general characteristics and the incidence of HPV infection and relevant disease in each age cohort by the exposure status. The categorical data will be described by frequency (proportion) and the Chi-squared test will be performed to show the group difference. The continuous data will be described by mean (standard deviation), and non-normal distribution continuous data will be described by median (quantiles), and then the ANOVA or Wilcoxon rank-sum test will be performed to compare the difference across age groups. Also, the Kaplan–Meier method will be used to draw the survival graph, based on the actual number of cases of each endpoint.

The Logistic Regression will be performed to analyze the impact on HPV 16/18 infection, and HPV 31/33/45 infection in each birth cohort 3 years after vaccination, after adjusting the confounders such as the demographic characteristics, history of sexual behavior, personal behavior history, etc. The vaccine impact will be calculated by using (1-Relative Risk) and its 95% CI.

Stratification analysis will be conducted by frequency of sexual activity, numbers of sexual partners, extramarital sexual activity, family annual income, education level, and cervical cancer screen history. The interaction term between the above factors and HPV vaccination will be introduced into the Logistic regression model, and the differences between the model with interaction term and the model without interaction term will be compared, so as to evaluate the interaction between different factors and HPV vaccination.

### Ethics and dissemination

The protocol for this study has been approved by the Peking University Institutional Review Board, which have accepted responsibility for supervising all aspects of the study (approval No: IRB00001052-22,014). All subjects and/or their LARs who agree to participate in the study will sign informed consent. Only the researchers associated with the study and the Ethics Committee would have access to the research data. The results of this study will be presented at both national and international conferences and be considered for publication in a peer-reviewed scientific journal. All the results presented in this study will be of group data; therefore, individual participants will not be identifiable. This study has been registered on *clinicaltrials.gov* (NCT05341284).

### Patient and public involvement

No subjects or their LARs has been or will be involved in the development of the plan for designing, conducting, reporting or implementing this study.

## Discussion

In order to eliminate cervical cancer during the twenty-first century, WHO recently launched a 90–70-90 target that advocated 90% of girls to be fully vaccinated with HPV vaccines by age 15 years, 70% of women to be screened with a high-performance test, and 90% of women identified with cervical disease received relevant treatment [7]. China is also striving to eliminate cervical cancer through the above methods, of which launching campaigns that promote the vaccination is an important component. Therefore, it is of great value to evaluate the impact of HPV vaccine in China, under the real-world setting. However, in China, only a few women have undergone cervical cancer screening before or just after the vaccination, and it is unclear if they will comply with regular screening later in their life. In this circumstance, it is challenging to acquire sufficient data to evaluate the HPV vaccination impact from a certain baseline and through a certain period of follow-up. Thus, although YRHIP is a high-quality regional database, the existing data are not sufficient to evaluate the impact of HPV vaccination on females aged under 35. This study, therefore, aims to establish prospective birth cohorts to describe the occurrence of HPV infection and related diseases among females in Yinzhou district, and to measure *Cervarix*’s impact in Chinese females.

### Strengths of the study

Our study has two main strengths. Firstly, this study will use the multi-age birth cohort design, and adjust the age confounders in multi-factor analysis to evaluate the impact of *Cervarix* in women of different ages. Previous studies have shown that the age of the HPV vaccination is one of the most important factors affecting its effectiveness [23]. Another strength is the combination of prospective cohort with the YRHIP. Monitoring outcomes using registry database was demonstrated to be a feasible and reliable way to evaluate the effectiveness of HPV vaccine [25, 26]. A prospective cohort study is used to match HPV vaccination with cervical cancer screening. At the same time, by combining the prospective cohort with real-world database, we are able to continually monitor the HPV related diseases after this study period, which will allow us to obtain the information on long-term impact of HPV vaccine. In addition, all the data collected from this study will be recorded in the YRHIP and contribute to the feasibility and capacity for the future HPV vaccine effectiveness surveillance.

### Feasibility and limitation

Despite the above strength, this study will still be facing some limitations and challenges in feasibility. First, the long recruitment period may face more uncertainties, such as the possible shortage of targeting vaccines and public health crisis like COVID-19, etc. If these uncertainties happen, it will eventually extend the whole study period and increase the risk of not completing the study in a timely and high-quality manner. Second, the number of drop-out participants in non-exposed group may lower the statistical power. It is planned that the administration of HPV vaccines in non-exposed group should be excluded during the follow up. Although subjects will be confirmed to be not having the intention to get HPV vaccination in the following 36 months when being recruited into the non-exposed group, there will still be possibility for them to get HPV vaccine. If this unexpected expose happens in large scale because of policy alteration such as the expanded HPV vaccination campaign in local area, this scenario may lead to the insufficient statistical power of final association analyses. However, with the high coverage of HPV vaccines, the alternative study design such as test-negative design may be able to be conducted. Since China is now making great effort to popularize different types of HPV vaccines, it is possible that a nonnegligible number of participants in non-exposure group will get vaccinated by quadrivalent and 9-valent vaccines. Thus, it is valuable to conduct post-hoc analysis by valent in such case. In addition, information on demographic characteristics, menstruation and obstetrical histories, history of sexual behavior, personal behavior history, history of disease and pathogen infection, medication history, and family history are collected using self-administered questionnaires, which will introduce recall bias and reporting bias.

This is the first prospective cohort study in China to assess the impact of HPV vaccine on HPV infection and related cervical diseases in females aged 9–45 years, which makes up for the lack of evidence in Chinese females. The results of this study will provide more evidence to understand the impact of bivalent HPV vaccination in Chinese females, and make a contribution to increasing HPV vaccination and cervical cancer screening coverage in China.

## Data Availability

The data that support the findings of this study are available from YRHIP but restrictions apply to the availability of these data, which were used under license for the current study, and so are not publicly available. Data are however available from the authors upon reasonable request and with permission of YRHIP.

## Abbreviations

HPV: Human papilloma virus
DNA: Deoxyribonucleic acid
PCR: Polymerase chain reaction
TCT: Thinprep cytologic test
YRHIP: Yinzhou Regional Health Information Platform
WHO: World Health Organization
CDC: Center for Disease Control and Prevention
ASC-US: Atypical squamous cells of undefined significance
AGC: Atypical glandular cells
ASC-H: Atypical squamous cells, cannot exclude for highly squamous intraepithelial lesions
LSIL: Low-grade squamous intraepithelial lesion
HSIL: High-grade squamous intraepithelial lesion
(CIN) 1, CIN2, CIN3: Cervical Intraepithelial Neoplasia
SCC: Squamous cell carcinoma
AIS: Adenocarcinoma in situ
ADC: Adenocarcinoma
LAR: Legally Acceptable Representative
